# L-Dopa response to Cortical Dysfunctions, health related quality of life and Fatigue Severity in Idiopathic Parkinson’s disease

**DOI:** 10.12669/pjms.344.14753

**Published:** 2018

**Authors:** Amara Gul, Javed Yousaf

**Affiliations:** 1 Dr. Amara Gul, PhD. Department of Applied Psychology, The Islamia University of Bahawalpur, Bahawalpur. Pakistan; 2 Javed Yousaf, M.Phil. Department of Applied Psychology, The Islamia University of Bahawalpur, Bahawalpur. Pakistan

**Keywords:** Parkinson’s Disease, Health-related quality of life, Cognition, Chronic Fatigue

## Abstract

**Objectives:**

To determine (i) levodopa (L-Dopa) responsiveness on cortical functions, health related quality of life and fatigue severity (ii) relationship between cortical functions, health related quality of life and fatigue severity post L-Dopa treatment of patients with idiopathic Parkinson’s Disease (I-PD).

**Methods:**

Participants included 50 patients diagnosed with I-PD who were attending Civil and Bahawal Victoria Hospital, Bahawalpur, Pakistan during May 2016 to July 2017 and 50 healthy individuals (HI) took part in the study. Participants completed Cortical Function Assessment, Parkinson’s Disease Questionnaire and Fatigue Severity Scale. Patients were tested twice on these measures: pre and post- L-Dopa treatment.

**Results:**

Patients with I-PD showed cortical functioning deficits, deteriorated health related quality of life and experience of severe fatigue, in contrast with HI. There was significant improvement in cortical functioning and quality of life while reduction in fatigue severity was observed after three months of L-Dopa medication in I- PD patients. Higher cortical functioning deficits correlated with deteriorated health related quality of life and severe fatigue. Cortical functioning was a significant predictor of health related quality of life and fatigue severity.

**Conclusion:**

L-Dopa is an effective treatment for cortical dysfunctions, health related quality of life and fatigue in I-PD. Cortical functioning is a significant marker of quality of life and fatigue in patients with I-PD.

## INTRODUCTION

Parkinson’s Disease (PD) is the second most common neurodegenerative disorder affecting 9 million people by 2030 in most populous nations of the world.^1^ Idiopathic Parkinson’s Disease (I-PD) is characterized by rigidity, tremor, and a kinesia due to several pathological mechanisms: activated microglia as evident in increased levels of [11C](*R*)-PK11195 binding in cortical and subcortical regions compared to normal controls in Positron Emission Tomography^2^, vulnerability of cortical areas and subcortical grays by formation of proteinaceous inclusion bodies beginning from dorsal motor nucleus in brain stem projecting upwards to mid brain, fore brain reaching cerebral cortex deteriorating limbic, autonomic and motor systems^3^, continuous local presence of neurotoxin until death in the form of nigral Glutathione transferase activity and total glutathione deficiency as consequent of overutilization in response to oxidative stress^4^, loss of corticocortical projection neurons, and striatal dopamine deficiency which contribute to cognitive deficits.^5^ Levodopa (L-Dopa) is an effective initial dopaminergic treatment for improvement of PD symptoms. In Pakistan, PD patients are given L-dopa as a mono-therapy or with combination of other pharmacological agents such as benserzide, anticholinergics etc.^6^ A recent study with Pakistani population showed high prevalence of cognitive deficits in patients with PD.^7^ Few studies have shown L-dopa beneficial effects on motor function, alertness, cognitive and neuropsychological performance of patients with PD.^8^,^9^

There is a missing link in previous literature about L-dopa response to cortical functioning, health related quality of life and fatigue severity in patients with I-PD. Therefore, the present study was designed to examine:

1. Effectiveness of L-Dopa on cortical functioning, health related quality of life and fatigue severity in patients with I-PD.
2. Relationship between cortical functioning, health’s related quality of life and fatigue severity in I-PD patients.

It was hypothesized:

1. I-PD patients would show deteriorated cortical functioning, declined health related quality of life and experience of severe fatigue in contrast with HI.
2. Compared with baseline performance, patients with I-PD would show improved cortical functioning, quality of life and reduced severity of fatigue post L-Dopa treatment.
3. Cortical functioning deficits would correlate with lesser quality of life and greater fatigue severity.
4. Cortical functioning would significantly predict quality of life and fatigue severity.

## METHODS

Fifty patients diagnosed with I-PD at Civil and Bahawal Victoria Hospital, Bahawalpur, Pakistan during May 2016 to July 2017 and 50 healthy individuals (HI) participated in the study. All participants were screened for:

1. Dementia using The Rowland Universal Dementia Assessment Scale^10^, score below 23/30 reflects dementia.
2. Depression through Geriatric Depression Scale-short form^11^ (score>5).

Inclusion criterion for patients was as follows:

1. Diagnosis of I-PD.
2. Stable on L-Dopa doses for at least 3 months.
3. Not taking any dopamine agonist, antidepressants, anticholinergic medication.
4. No present complaints or history of any psychiatric disorder.
5. No present illness or history of neurological disorder except PD.

Cortical Function Assessment^12^ (CFA) was used to assess cortical functions: sensory extinction, dictation, naming, writing, repetition, drawing, and stereognosis. It is a simple, bedside, 10-item clinical assessment. The completion time is approximately 10 minutes. Total score range is 0-10. Lower scores show cortical function deficits. The test has good psychometric characteristics.

Parkinson’s Disease Questionnaire^13^ (PDQ) was used to assess health related quality of life in PD patients. It is a 39 item questionnaire to examine eight dimensions of functioning: emotional well-being, activities of daily living, mobility, social support, stigma, communication, cognition, bodily discomfort on 4 point response categories (Total score range= 0-156). Higher score shows deteriorated quality of life. The test is sensitive to evaluate PD related dysfunctions.

Fatigue Severity Scale^14^ (FSS) was used to measure chronic fatigue. It is a 9-item scale scored on 7-point response categories (1=strongly disagree to 7= strongly agree). Mean score on items is used as an index of fatigue severity. Higher score shows greater fatigue severity.

### Procedure

The study was approved by board of studies of The Islamia University of Bahawalpur. After obtaining informed consent from HI, patients and their caregivers, a trained psychologist administered psychological testing. Participants and psychologist were both blinded to objectives of the study. All participants completed CFA, PDQ and FSS. HI has single testing session whereas patients had two testing sessions. First session was conducted at the time of I-PD diagnosis. Second session was conducted after at least three months of L-dopa treatment. Testing on all mentioned measures were completely randomized across patients to avoid practice effects.

### Statistical Analyses

Repeated measures analysis of variance (ANOVA) was conducted with CFA scores 3 (controls vs. patient’s pretreatment vs. patient’s post-treatment) as within subjects factor. Separate ANOVA was conducted with scores on FSS as within subjects repeated factor 3 (controls vs. patient’s pretreatment vs. patient’s post-treatment). ANOVA was conducted with scores on PDQ as within subject repeated factor 3 (controls vs. patient’s pretreatment vs. patient’s post-treatment). Scores on subscales of PDQ 8 (emotional well-being vs. activities of daily living vs. mobility vs. social support vs. stigma vs. communication vs. cognition vs. body discomfort: within subjects) x Group-3 (controls vs. patient’s pretreatment vs. patient’s post-treatment: between subjects) were analyzed through repeated measures ANOVA. Bivariate correlations will be used to analyze association between post-treatment scores on CFA, FSS and PDQ. Separate regression analysis was conducted to assess CFA scores (independent variable) as predictor of performance on PDQ and FSS (dependent variables) post L-Dopa treatment.

## RESULTS

Participants had age range between 45 to 65 years. There was no significant difference on age between both groups I-PDP (M± SD 56.06±5.76) HI (M± SD 57.12±5.09) *t* (49)=1.02, p=0.30. The ratio of male and female was 50% in each group (25: 25). Disease duration (years) for I-PDP was M± SD 3.52±0.81 and L-dose daily (mg) was M± SD 486.80±54.73. There were significant within subject differences on cortical functioning *F* (2, 98) = 1174.33, p<0.001, ηp2=0.96, quality of life *F*(2, 98)=4949.96, p<0.001, ηp2=.99, and FSS*F* (2, 98)=1028.81, p<0.001, ηp2=0.95. Patients with I-PD showed cortical functioning deficits, deteriorated quality of life and fatigue severity in contrast with HI. Post-treatment assessment showed improvement in cortical functioning, quality of life and reduced fatigue severity. Repeated measures ANOVA to assess differences between groups on subscales of PQD showed significant main effects of PDQ *F* (7, 147)=11689.77, p<0.001, ηp2=0.98, Group-*F* (2, 147)= 2546.90, p<0.001, ηp2=0.97 and interaction between PDQ x Group-*F* (14, 147)=3037.01, p<0.001, ηp2=0.97 (Table-I). This result revealed that in contrast with HI, I-PD patients showed deterioration in all areas of quality of life while post-treatment scores showed significant improvement in emotional well-being, activities of daily living, mobility, social support, stigma, communication, cognition, and body discomfort. Post-treatment scores on CFA were inversely correlated with scores on FSS (r=-0.51, p<0.001) and PDQ (r= -0.80, p<0.001) in patients with I-PD. Regression analysis showed cortical functioning as significant predictor of fatigue severity R^2^=0.27, *F*(1, 49)=17.73, p<0.001, β=-0.51, t=-4.21, p<0.001and quality of life R^2^=0.64, *F*(1, 49)=87.56, p<0.001, β=-0.80, t=-9.35, p<0.001 in patients with I-PD post L-Dopa treatment.

**Table-I:** Clinical characteristics of sample.

	95% CI		
	M±SD	Lower bound	Upper bound
**Cortical function assessment**			
I-PDP pretreatment	2.82±0.69	2.62	3.01
I-PDP post-treatment	6.36± 0.82	6.12	6.59
HI	9.68±0.47	9.54	9.81
**Fatigue severity scale**			
I-PDP pretreatment	6.02±0.65	5.83	6.20
I-PDP post-treatment	4.14±0.45	4.01	4.26
HI	1.14±0.35	1.04	1.24
**Parkinson’s disease questionnaire**			
I-PDP pretreatment	132.92±12.57	129.34	136.49
I-PDP post-treatment	86.36±8.58	83.92	88.79
HI	7.02±2.63	6.27	7.77
**Emotional-well-being**			
I-PDP pretreatment	22.56±0.92	22.33	22.79
I-PDP post-treatment	18.58±0.92	18.35	18.81
HI	0.76±0.55	0.53	0.99
**Activities of daily living**			
I-PDP pretreatment	22.46±0.90	22.32	22.68
I-PDP post-treatment	18.54±0.90	18.31	18.76
HI	0.84±0.58	0.61	1.06
**Mobility**			
I-PDP pretreatment	37.16±1.62	36.81	37.50
I-PDP post-treatment	30.34±1.22	29.99	30.68
HI	0.80±0.69	0.45	1.14
**Social support**			
I-PDP pretreatment	8.48±1.97	8.10	8.85
I-PDP post-treatment	3.30±1.11	2.92	3.67
HI	0.98±0.62	0.60	1.35
**Stigma**			
I-PDP pretreatment	12.44±2.11	11.97	12.90
I-PDP post-treatment	4.30±1.79	3.83	4.76
HI	0.82±0.71	0.35	1.28
**Communication**			
I-PDP pretreatment	8.72±2.00	8.33	9.10
I-PDP post-treatment	3.38±1.14	2.99	3.76
HI	1.02±0.65	0.63	1.40
**Cognition**			
I-PDP pretreatment	12.48±2.14	12.02	12.93
I-PDP post-treatment	4.50±1.65	4.04	4.95
HI	0.82±0.71	0.36	1.27
**Body discomfort**			
I-PDP pretreatment	8.62±2.08	8.21	9.02
I-PDP post-treatment	3.42±1.24	3.01	3.82
HI	0.98±0.62	0.57	1.38

*Note:* Read CI: confidence interval, M±SD: Mean ± Standard deviation, HI: Healthy individuals (n=50),

I-PDP: Idiopathic Parkinson’s disease Patients (n=50).

## DISCUSSION

There were few important results.

1. Patients with I-PD had cortical functioning deficits, deteriorated health related quality of life and fatigue severity in contrast with HI.
2. After three months of L-Dopa treatment, there was significant improvement in cortical functioning and health related quality of life in I-PD patients. Reduction in fatigue severity was also observed in response to L-Dopa therapy in patients with I-PD.
3. Cortical functioning was negatively correlated with quality of life and fatigue severity.
4. Cortical functioning was a significant predictor of fatigue severity and quality of life in I-PD patients.

Presence of cognitive deficits in patients with PD have been well-documented in previous studies in Pakistani and Western population.^6^-^8^ To be best of to our knowledge, there are no studies which examined cortical functions in this patient group. Thus, results of the present study highlighted cortical function deficits in patients with I-PD. Cortical functioning deficits in patients with I-PD have several pathological factors such as increased levels of [11C](*R*)-PK11195 in cortical/ subcortical regions, susceptibility of cortical/subcortical grays due to development of proteinaceous inclusion bodies starting from brain stem bulging to cerebral cortex weakening limbic, autonomic and motor systems, constant presence of neurotoxin as nigral Glutathione transferase activity and total glutathione deficiency as a result of overutilization in response to oxidative stress, loss of corticocortical projection neurons in motor cortex, and dopamine loss.^2^-^5^ These pathological features contribute to cognitive deficits in patients with PD.

Previous studies with PD patients not only showed that cognitive decline had greater impact on quality of life rather severity of cognitive decline contributed to poor quality of life.^15^,^16^ Correlation analysis in the present study showed that higher cortical functioning deficits were associated with deteriorated health related quality of life and experience of severe fatigue in patients with I-PD. Cortical functioning was significant predictor of fatigue severity and health related quality of life in I-PD patients. These results are consistent with previous findings of deteriorated quality of life with cognitive decline in such patients. Whilst existent studies in literature have not assessed relationship between cortical functioning, health related quality of life and fatigue severity, thus the present finding is an addition to the literature.

Scarce data have shown that three months treatment of L-dopa is effective in reducing cognitive impairment in PD patients but studies have not assessed efficacy of L-dopa on cortical functions. Result of the present study showed that L-Dopa can bring positive changes in cortical functioning, quality of life and experience of fatigue in patients with I-PD. This finding is consistent with previous studies showing improvement in non-motor symptoms, for instance memory problems in PD patients.^8^,^9^ Findings of the current study have implications in better patient care and rehabilitation. Cortical functioning must be assessed at initial stages of treatment in I-PD patients to prevent further deterioration. This study highlighted cortical functioning as a marker of quality of life and fatigue severity in patients with I-PD.

### Limitations

Long term effects of L-Dopa on cortical functioning, quality of life and fatigue must be studied which constitute a limitation of the present study since the possibility that long-term use of L-Dopa might have adverse effects on these variables. Future studies must compare impacts of short term and long term L-Dopa therapy on cortical functioning, quality of life and fatigue in I-PD patients.

### Author`s Contribution

**AG** conceived the study, designed, analyzed data, manuscript writing.

**JY** did literature review, collected data.
